# Suppression of the auxin response pathway enhances susceptibility to *Phytophthora cinnamomi* while phosphite-mediated resistance stimulates the auxin signalling pathway

**DOI:** 10.1186/1471-2229-14-68

**Published:** 2014-03-20

**Authors:** Leila Eshraghi, Jonathan P Anderson, Nader Aryamanesh, Jen A McComb, Bryan Shearer, Giles St J E Hardy

**Affiliations:** 1 Centre for Phytophthora Science and Management, School of Veterinary and Life Sciences, Murdoch University, South Street, Murdoch, WA 6150, Australia; 2 CSIRO Plant Industry, Centre for Environment and Life Sciences, Private Bag 5, Wembley, WA 6913, Australia; 3 School of Plant Biology, Faculty of Science, The University of Western Australia, 35 Stirling Highway, Crawley, WA 6009, Australia; 4 The University of Western Australia, Institute of Agriculture, 35 Stirling Highway, Crawley, WA 6009, Australia; 5 Science Division, Department of Environment and conservation, Kensington, WA 6983, Australia

**Keywords:** Abscisic acid, Indole-3-acetic acid (IAA), Phosphate starvation response (PSR), TIBA, Ubiquitin proteasome pathway (UPP)

## Abstract

**Background:**

*Phytophthora cinnamomi* is a devastating pathogen worldwide and phosphite (Phi), an analogue of phosphate (Pi) is highly effective in the control of this pathogen. Phi also interferes with Pi starvation responses (PSR), of which auxin signalling is an integral component. In the current study, the involvement of Pi and the auxin signalling pathways in host and Phi-mediated resistance to *P. cinnamomi* was investigated by screening the *Arabidopsis thaliana* ecotype Col-0 and several mutants defective in PSR and the auxin response pathway for their susceptibility to this pathogen. The response to Phi treatment was also studied by monitoring its effect on Pi- and the auxin response pathways.

**Results:**

Here we demonstrate that *phr1*-*1* (phosphate starvation response 1), a mutant defective in response to Pi starvation was highly susceptible to *P. cinnamomi* compared to the parental background Col-0. Furthermore, the analysis of the *Arabidopsis tir1*-*1* (transport inhibitor response 1) mutant, deficient in the auxin-stimulated SCF (Skp1 − Cullin − F-Box) ubiquitination pathway was also highly susceptible to *P. cinnamomi* and the susceptibility of the mutants *rpn10* and *pbe1* further supported a role for the 26S proteasome in resistance to *P. cinnamomi*. The role of auxin was also supported by a significant (*P* < 0.001) increase in susceptibility of blue lupin (*Lupinus angustifolius*) to *P. cinnamomi* following treatment with the inhibitor of auxin transport, TIBA (2,3,5-triiodobenzoic acid). Given the apparent involvement of auxin and PSR signalling in the resistance to *P. cinnamomi*, the possible involvement of these pathways in Phi mediated resistance was also investigated. Phi (especially at high concentrations) attenuates the response of some Pi starvation inducible genes such as *AT4*, *AtACP5* and *AtPT2* in Pi starved plants. However, Phi enhanced the transcript levels of *PHR1* and the auxin responsive genes (*AUX1*, *AXR1*and *AXR2*), suppressed the primary root elongation, and increased root hair formation in plants with sufficient Pi.

**Conclusions:**

The auxin response pathway, particularly auxin sensitivity and transport, plays an important role in resistance to *P. cinnamomi* in *Arabidopsis*, and phosphite-mediated resistance may in some part be through its effect on the stimulation of the PSR and auxin response pathways.

## Background

The plant pathogen *Phytophthora cinnamomi* causes considerable damage to agriculture, horticulture and native plant communities worldwide [1-6]. Phosphite (Phi), an analogue of phosphate (Pi) is a salt of phosphorous acid (H_3_PO_3_) and is highly effective in controlling *P. cinnamomi*[3,5,7,8]. However, little is known about the mode of action of Phi on induction of resistance to this pathogen. Understanding the molecular mechanisms underlying plant―*Phytophthora cinnamomi* interactions and the effect of Phi on these interactions may allow the design of strategies to improve disease resistance or the more effective use of Phi.

Resistance to potential pathogens depends on interaction between different plant defence signalling pathways such as those regulated by the phytohormones salicylic acid (SA), jasmonic acid (JA), ethylene (ET), abscisic acid (ABA), and auxin [9]. Synergistic and antagonistic interactions between different signalling pathways induced by phytohormones and their effect on induction of resistance to biotrophic or necrotrophic pathogens have been well documented [9-14].

Phi is believed to mimic Pi and interferes with the manifestation of a wide range of biochemical and developmental Pi starvation responses (PSR) in *Arabidopsis thaliana* and other plant species [15-18]. Pi status is very important for determining root architecture mediated through the auxin signalling pathway and auxin signalling is required for the full Pi starvation response [19-23].

Auxin signalling is mediated largely by the SCF^TIR1^ E3 ubiquitin ligase complex (UPP complex) that accelerates AUXIN/INDOLE-3-ACETIC ACID (AUX/IAA) repressor protein degradation in response to auxin [24-27]. The AUX/IAA repressor proteins are recognized and ubiquitinated by a ubiquitin-conjugation cycle involving an E1 (AXR1 and ECR1), an E2 (RCE1), and the SCF^TIR1^ E3, which consists of a Cullin-CUL1, the SKP1-ASK1, RBX1, and the F-Box protein TIR1 (TRANSPORT INHIBITOR RESPONSE1) [28]. Pi modulates auxin sensitivity via the auxin receptor TIR1 and Pi starvation increases the expression of the *TIR1* gene in *Arabidopsis* seedlings leading to degradation of AUX/IAA repressors and activation of downstream auxin responses [24,25]. SGT1B protein functions in SCF-TIR1 mediated degradation of AUX/IAA proteins [29-31] and interacts with *RAR1*; a component of R-gene-mediated resistance [31-33]. Both RAR1 and SGT1B interact with COP9 which is involved in protein degradation by the 26S proteasome [32,34]. Furthermore, auxin has been implicated in the induction of resistance against some necrotrophic pathogens including *Plectosphaerella cucumerina* and *Botrytis cinerea*[9,35].

In conclusion, the involvement of the auxin signalling pathway in plant defence and Pi signalling, together with interference of Phi in Pi homeostasis and PSR indicates a possible involvement of the auxin signalling pathway in resistance of some *Arabidopsis* accessions to *P. cinnamomi*, previously reported to possess a predominantly necrotrophic lifestyle [36] and in Phi mediated resistance. The objectives of this study were to examine the potential involvement of Pi and auxin signalling pathways in resistance to *P. cinnamomi* by screening the mutants defective in PSR, auxin and ubiquitin signalling pathways for their susceptibility to this pathogen and to investigate whether Phi induces resistance to *P. cinnamomi* by manipulating the PSR and auxin signalling pathways by studying the effect of Phi on Pi signalling and the importance of their concomitant effect on activation/suppression of the auxin response pathway in relation to PSR. The mechanism of action of Phi was further examined by investigating its effect on morphological PSR responses and analysis of Pi starvation gene expression following Phi treatment under Pi sufficient and deficient conditions and in auxin and ABA response modulation of Pi signalling involving auxin.

## Results

### Resistance to *P. cinnamomi* was reduced in the Pi starvation response mutant

**Figure 1:**
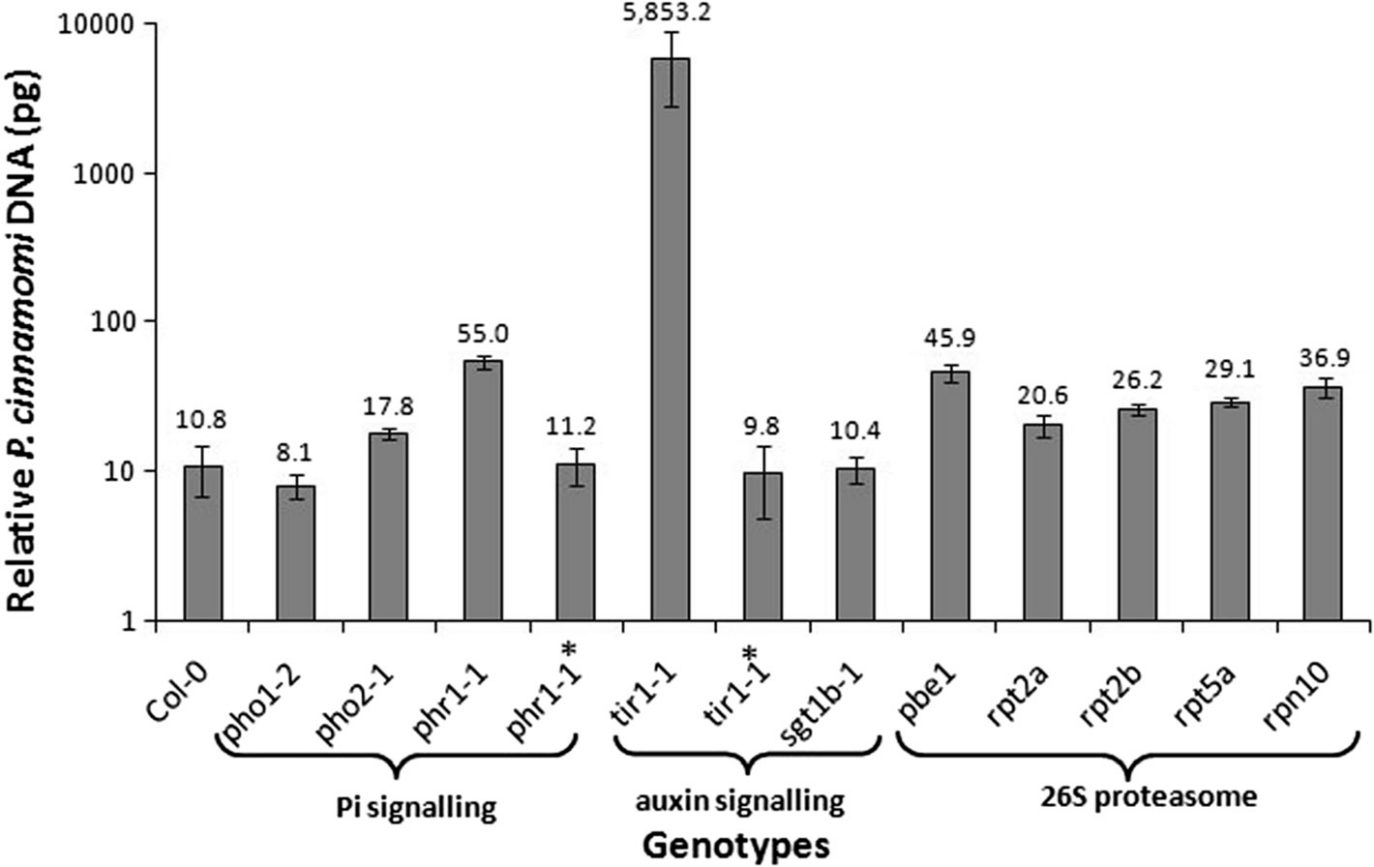
***Phytophthora cinnamomi*response in several mutants of*Arabidopsis thaliana*defective in auxin signalling, phosphate signalling and 26S proteasome subunits.** Quantitative PCR (QPCR) assessment of *Phytophthora cinnamomi* biomass (pg DNA per sample) 72 h after inoculation of attached leaves of *Arabidopsis thaliana* ecotype Colombia (Col-0) and several *Arabidopsis* mutants defective in Pi signalling (*pho1*-*1*, *pho2*-*1*, and *phr1*-*1*), the auxin signalling pathway (*tir1*-*1*and *sgt1b*-*1*), and 26S proteasome subunits (*pbe1*, *rpt2a*, *rpt2b*, *rpt5a* and *rpn10*). Bars represent the mean and standard error from five replicates each consisting of four infected leaves. One way ANOVA indicated a significant (*P* < 0.001) difference between genotypes. *LSD* (5%) was 8.92. *phr1*-1* = *phr1*-*1* mutant complemented with *PHR1* gene (AT4G28610) and *tir1*-1* = *tir1*-*1* mutant complemented with *TIR1*gene (AT3G62980).

To investigate whether Pi signalling affects the response of *Arabidopsis* to *P. cinnamomi*, ecotype Col-0 and several PSR mutants *phr1*-*1*, *pho2*-*1*, and *pho1*-*2* were screened for their susceptibility to the pathogen and the level of infection was determined quantitatively according to Eshraghi et al. [37]. The QPCR analysis of infection showed significantly (*P* < 0.001) greater *P. cinnamomi* biomass in the *phr1*-*1* mutant compared to that in its wild background Col-0 (Figure 1) suggesting a role of Pi signalling in resistance to *P. cinnamomi*. Furthermore, transferring the cloned *PHR1* gene into the susceptible *phr1*-*1* mutant restored resistance to the level observed in the parental background Col-0 (Figure 1) confirming that the mutant was susceptible due to loss of PHR1 function. The *PHR1* gene contributes to downstream Pi signalling by regulating the expression of Pi responsive genes [38-40] and the *phr1* mutant is defective in Pi signalling [38]. PHO1 and PHO2 both act downstream of the *PHR1* transcription factor to control the local uptake or transport of Pi [41-44]. The *pho2*-*1* and *pho1*-*2* mutants did not show significant (*P* > 0.05) increase in their susceptibility to *P. cinnamomi* compared to Col-0 (Figure 1).

### The SCF^TIR1^ complex is involved in resistance to *P. cinnamomi* infection

The high susceptibility of the *phr1*-*1* mutant in the current study, combined with the role of the auxin signalling pathway in the PSR and plant resistance [19,35] suggested a possible involvement of auxin signalling in resistance to *P. cinnamomi*. QPCR assessment of pathogen biomass showed that *tir1*-*1*; an auxin response mutant deficient in the auxin-stimulated SCF (Skp1 − Cullin − F-Box) ubiquitination pathway [45-48] was highly susceptible to *P. cinnamomi* (Figure 1). Furthermore, transferring the cloned *TIR1* gene into the *tir1*-*1* mutant restored resistance to the level observed in the parental background Col-0 (Figure 1) and confirmed that susceptibility was due to loss of TIR1 function in the mutant.

Since *Arabidopsis* SGT1B contributes to the auxin response controlled by the SCF^TIR1^ complex [30,45] and functions in plant disease resistance signalling [29], we investigated whether mutations in SGT1B also affect resistance to *P. cinnamomi*. QPCR analysis showed no significant (*P* > 0.05) differences in susceptibility of *sgt1b*-*1* in comparison to its wild parental background Col-0 (Figure 1) suggesting that SGT1B does not contribute to SCF-related processes in resistance to *P. cinnamomi*.

### The 26S proteasome subunits are involved in resistance to *P. cinnamomi*

The 26S proteasome is involved in the degradation of AUX/IAA proteins and consequently activation of auxin responsive genes [49]. In the present study, several *Arabidopsis* mutants defective in 26S proteasome subunits (*pbe1*, *rpt2a*, r*pt2b*, *rpt5a* and *rpn10*) were screened for their susceptibility to *P. cinnamomi*. The analysis of infection revealed that the *Arabidopsis* mutants *pbe1*, a knockout mutant for 20S proteasome [50] and *rpn10* with reduced auxin sensitivity [51] were both significantly (*P* < 0.001) more susceptible to *P. cinnamomi* compared to their parental background Col-0, with 45.9 and 36.9 pathogen biomass (pg DNA), respectively (Figure 1). Furthermore, the susceptibility of the *Arabidopsis* 26S proteasome subunit mutants *rpt5*a (29.1 pg), *rpt2a* (20.6 pg), and *rpt2b* (homologue of *rpt2a*; 26.2 pg) was significantly (*P* < 0.001) higher compared to that in their background Col-0 (10.8 pg, Figure 1).

### Inhibition of auxin transport by TIBA treatments enhanced *P. cinnamomi* infection

**Figure 2:**
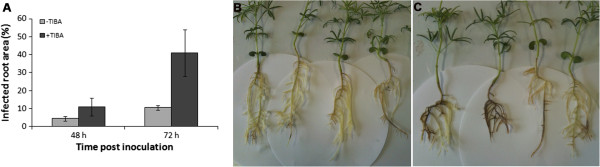
**Negative effect of auxin transporter inhibitor (TIBA) on*Phytophthora cinnamomi*resistance in lupin.** The effect of TIBA; an auxin transport inhibitor on lesion development in *Lupinus angustifolius* roots inoculated with *Phytophthora cinnamomi* mycelial plugs. **(A)** Percentage infected root area in TIBA-treated (+TIBA) and non-TIBA-treated (−TIBA) lupin roots 48 h and 72 h after inoculation. **(B)** and **(C)** show disease symptoms caused by *P. cinnamomi* infection in non-TIBA-treated **(B)** and TIBA-treated **(C)**. One way ANOVA indicated a significant (*P* < 0.001) difference between treatments.

The susceptibility of the *Arabidopsis* auxin response mutant *tir1*-*1*[35] suggested the involvement of auxin response pathway in the outcome of *A. thaliana*―*P. cinnamomi* resistance (Figure 1). To test this further, blue lupin (susceptible to *P. cinnamomi*) seedlings were treated with an auxin transport inhibitor, TIBA, and their susceptibility determined. For these studies we used blue lupin rather than *Arabidopsis* because of the large size of the root system and susceptibility to *P. cinnamomi* allowing clearer observation of potential differences. Infection in lupin seedling roots treated with TIBA was significantly (*P* < 0.001) greater than in non-treated plants 72 h after inoculation (Figure 2).

### Expression of Pi-and auxin signalling-related genes in response to Phi treatments

The relative expression ratios of the Pi responsive genes *AtPT2*, *AtACP5* and *AT4* (Additional file 1: Table S1) in Col-0 grown under Pi sufficient or Pi deficient conditions were analysed following Phi treatments (Table 1). The transcript levels of *AT4*, *AtPT2*, and *AtACP5* increased significantly (*P* < 0.001) in response to Pi deficiency in wild ecotype Col-0 (Table 1). In contrast, the transcript levels of the *AT4*, *AtPT2*, and *AtACP5* genes were greatly suppressed (55.6-fold, 8.9-fold, and 4.2-fold, respectively) when the Pi starved plants were treated with 2.5 mM Phi. Furthermore, the high level of Phi (20 mM) suppressed the up-regulation of *AT4* (1.1-fold), *ATPT2* (1.1-fold), and *AtACP5* (1.07-fold) in response to Pi deficiency to the levels observed in plants grown in Pi sufficient (control) conditions demonstrating an impact of Phi on the PSR (Table 1).

**Table 1:** **Expression analysis of phosphate starvation response** (**PSR**) **genes in*Arabidopsis thaliana*ecotype Columbia** (**Col**-**0**) **grown under phosphate** (**Pi**) **sufficient** (+**Pi**; **1.25 mM**) **and Pi deficient** (−**Pi**; **0 mM**) **conditions and subjected to different phosphite** (**Phi**) **treatments**

	**Normalized relative transcript level**						**Relative fold difference to control**				
	**+Pi**			**-Pi**			**+Pi**		**-Pi**		
**Gene**^**a**^	**0 mM Phi (control)**	**2.5 mM Phi**	**20 mM Phi**	**0 mM Phi**	**2.5 mM Phi**	**20 mM Phi**	**2.5 mM Phi**	**20 mM Phi**	**0 mM Phi**	**2.5 mM Phi**	**20 mM Phi**
*AT4*	0.261_± 0.005_	0.175 _± 0.013_	0.306 _± 0.033_	73.127_± 4.331_	14.512 _± 0.264_	0.290 _± 0.026_	0.7	1.2	280.4	55.6	1.11
*AtPT2*	0.449 _± 0.011_	0.662 _± 0.112_	0.527 _± 0.016_	12.537 _± 0.70_	3.979 _± 0.126_	0.499 _± 0.082_	1.5	1.2	27.9	8.9	1.11
*AtACP5*	0.429 _± 0.008_	0.560 _± 0.032_	0.432 _± 0.005_	8.354 _± 0.434_	1.803 _± 0.049_	0.459 _± 0.007_	1.3	1.0	19.5	4.2	1.07

The transcript level of genes was normalized based on the expression of actin 2 (ACT2) measured in the same samples and presented as both normalized relative transcript level (mean ± SE) and the factor of increase in transcription compared with the control (+Pi/0 mM Phi). Data represent the mean and standard error of four biological replicates per treatment each consisting of three plants.

^a^See Table 2.

**Figure 3:**
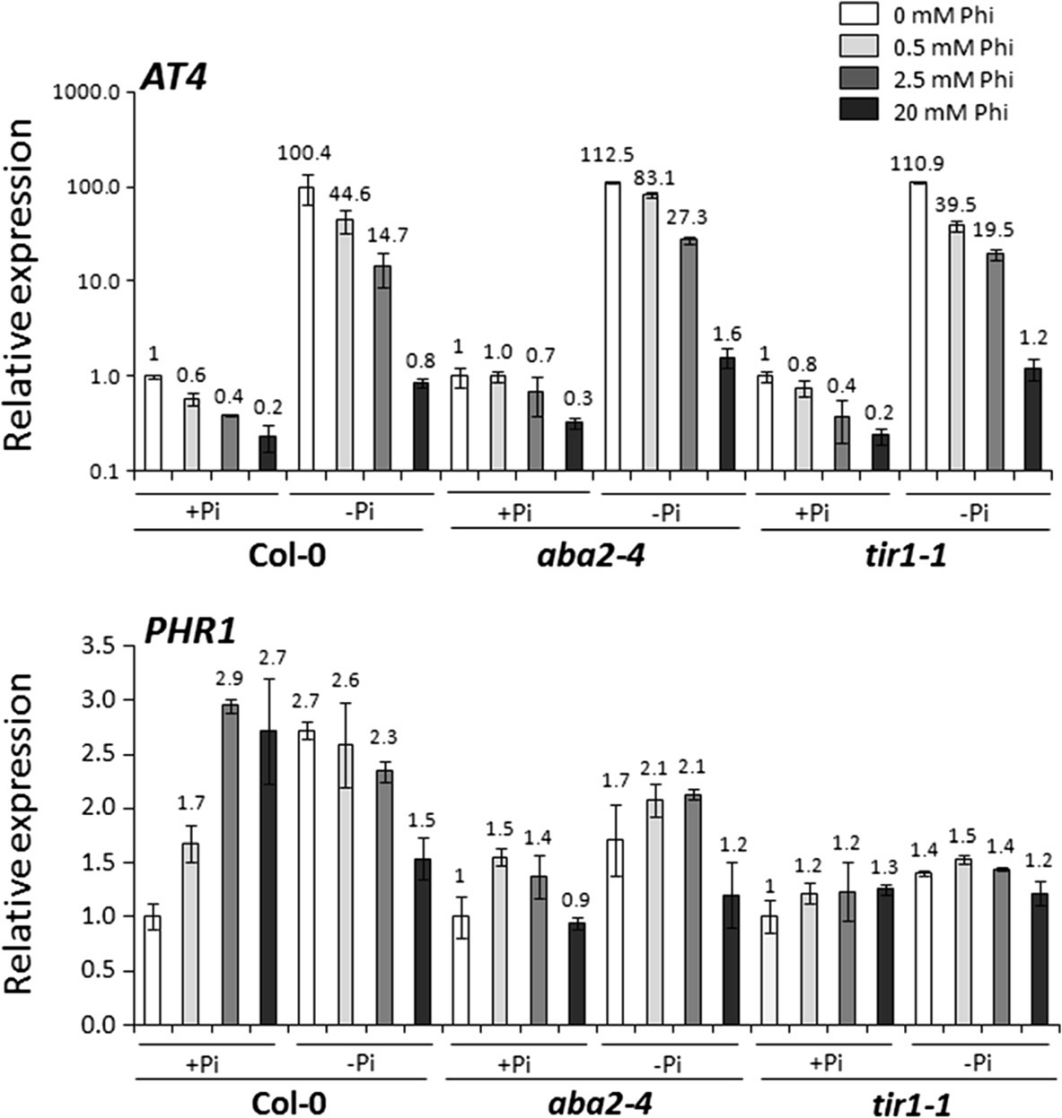
**Effect of phosphite on phosphate starvation responsive genes in Col-0,*aba2*-*4*and*tir1*-*1.*** Relative expression ratios of *AT4* and *PHR1* transcripts in *Arabidopsis thaliana* wild ecotype (Col-0) and *Arabidopsis* mutants *aba2*-*4* and *tir1*-*1* grown in phosphate (Pi) sufficient media (1.25 mM) for three weeks followed by a further five days growth in different Pi (1.25 mM Pi; +Pi and 0 mM Pi; −Pi) and phosphite (Phi) (0, 0.5, 2.5 and 20 mM) levels. The transcript levels in the mutant were normalized based on the expression of actin 2 (*ACT2*) measured in the same samples and presented relative to the normalized expression levels in non-Phi treated, Pi-sufficient grown Col-0. Bars present the mean and standard error from four replicates each consisting of three plants. The numbers on each bar indicate the fold change in gene transcript levels relative to corresponding Pi sufficient (+Pi), non-Phi-treated plants. One way ANOVA indicated a significant (*P* < 0.001) difference between genotypes. *LSD* (5%) for *AT4* and *PHR1* were 23.96 and 1.01, respectively.

Given the apparent involvement of the Pi and auxin signalling pathways in the resistance of Col-0, the interaction of these pathways with ABA signalling [42] and previous observations of the susceptibility of ABA signalling mutants by Eshraghi et al. [52], the effects of Phi treatment on PSR gene expression was investigated in Col-0, *aba2*-*4* and *tir1*-*1* plants (Figure 3). The transcript level of the *AT4* in Pi deficient, non-Phi-treated Col-0, *aba2*-*4* and *tir1*-*1* significantly (*P* < 0.05) increased 100-fold, 112-fold, and 111-fold, respectively compared to Pi sufficient Col-0. However, Phi treatments resulted in suppression of the *AT4* gene in all plants tested with the level of this suppression depended on the concentration of Phi applied (Figure 3).

In non-Phi-treated Col-0, the transcript level of *PHR1* (phosphate starvation response 1) in response to Pi deficiency increased (2.7-fold) and Phi treatments suppressed the transcript level of this gene with the highest suppression in 20 mM Phi-treated plants (Figure 3). Although Phi treatment in Pi deficient grown samples suppressed the expression of *PHR1*, the transcript level of this gene was induced in Col-0 samples grown in Pi sufficient media and the level of this induction depended on the concentration of Phi applied in this study (Figure 3). The expression of *PHR1* in response to Pi starvation was diminished in *aba2*-*4* and *tir1*-*1* mutants compared to that in Col-0 indicating the importance of *ABA2* and *TIR1* genes in induction of *PHR1*. Furthermore, Phi treatments had no significant (*P* > 0.05) effect on transcript level of the *PHR1* gene in either the *aba2*-*4* or *tir1*-*1* mutants suggesting that mutation in *ABA2* and *TIR1* genes may disrupt the Phi effect on Pi signalling.

The high susceptibility of the *Arabidopsis* mutant *tir1*-*1* (Figure 1) and the enhanced level of *P. cinnamomi* infection in roots of TIBA-treated lupins (Figure 2) showed that the auxin response pathway plays an important role in resistance to *P. cinnamomi*. In addition, the induction of *PHR1* gene by Phi in Col-0 samples grown in Pi sufficient media suggested that Phi induces PSR and loss of *PHR1* gene expression in the *tir1*-*1* mutant highlighted the possible induction of the auxin response pathway by Phi treatments. Therefore, we hypothesised that Phi mediated resistance to *P. cinnamomi* may be through induction of the auxin response pathway.

**Figure 4:**
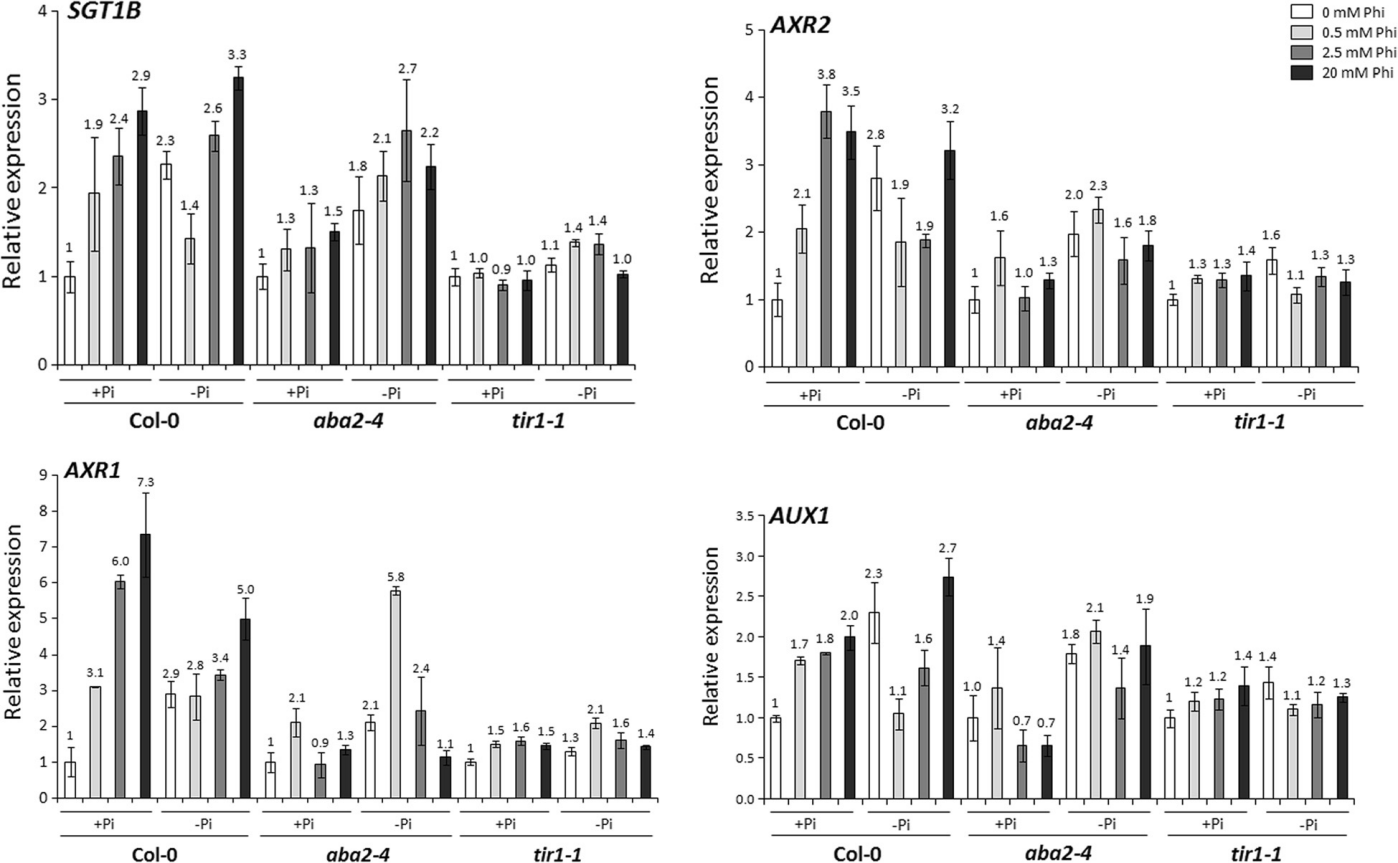
**Effect of phosphite on induction of auxin responsive genes.** Relative expression ratios of *AUX1*, *AXR1*, *AXR2* and *SGT1B* transcripts in *Arabidopsis thaliana* wild ecotype (Col-0) and mutants (*aba2*-*4* and *tir1*-*1*) grown in a phosphate (Pi) sufficient medium (1.25 mM) for three weeks following further five days growth in different Pi (1.25 mM Pi; +Pi and 0 mM Pi; −Pi) and phosphite (Phi) (0, 0.5, 2.5 and 20 mM) levels. The transcript levels in the mutant were normalized based on the expression of actin 2 (*ACT2*) measured in the same samples and presented relative to the normalized expression levels in non-Phi treated, Pi-sufficient grown Col-0. Data represent the mean and standard error of four replicates of three plants each. The numbers on each bar indicate the fold increase/decrease in transcript levels relative to corresponding Pi sufficient (+Pi), non-Phi-treated plants. ANOVA indicated a significant (*P* < 0.05) difference between treatments for all genotypes. *LSD* (5%) for *SGT1B*, *AXR1*, *AXR2*, and *AUX1* were 1.06, 1.25, 1.16, and 0.82 respectively.

To test this hypothesis, the concomitant effect of Phi and Pi on auxin signalling in Pi sufficient/Pi deficient grown Col-0, *aba2*-*4* and *tir1*-*1* mutants following 0, 0.5, 2.5 and 20 mM Phi treatments was assessed by measuring the relative expression ratios of auxin responsive genes, *AUX1*, *AXR1*, *AXR2* and *SGT1B* transcripts (Additional file 1: Table S1). In non-Phi-treated Col-0 seedlings, Pi deficiency increased the transcript levels of *AUX1* (2.3-fold), *AXR1* (2.9-fold), *AXR2* (2.8-fold) and *SGT1B* (2.3-fold) genes indicating activation of the auxin response pathway in Pi deficient plants (Figure 4). In Pi sufficient Col-0, Phi treatments (2.5 and 20 mM) induced significantly (*P* < 0.05) the transcript levels of *AUX1*, *AXR1*, *AXR2* and *SGT1B* genes and the level of this induction depended on the Phi concentrations used (Figure 4). Addition of Phi to Pi starved plants at 0.5 mM suppressed the enhanced transcript level of *AUX1* (from 2.3-fold to 1.1-fold); while, Phi treatments at 0.5 mM had no significant (*P* > 0.05) effect on the expression of *AXR1*, *AXR2* and *SGT1B* in Pi deficient Col-0 (Figure 4). However, application of Phi at higher concentrations (2.5 and 20 mM) to Pi starved plants had no significant (*P* > 0.05) effect on the expression of *AUX1*, *AXR1*, *AXR2* and *SGT1B* genes (Figure 4).

In Pi deficient, non-Phi-treated *aba2*-*4* mutant, the transcript levels of *AUX1*, *AXR1*, *AXR2* and *SGT1B* genes did not increase significantly (*P* > 0.05) compared to that in the Pi sufficient, non-Phi-treated *aba2*-*4* mutant (Figure 4) suggesting that PSR- responsive expression of the auxin responsive genes is reliant on ABA signalling. Furthermore, application of Phi to the *aba2*-*4* mutant grown under either Pi sufficient or Pi deficient conditions did not increase transcript level of auxin responsive genes (*AUX1*, *AXR1*, *AXR2* and *SGT1B*) with the exception of 0.5 mM Phi-treated plants for the *AXR1* gene (Figure 4). These results suggest that the Phi-mediated activation of auxin responsive genes may involve ABA signalling. In the *tir1*-*1* mutant, Pi starvation did not considerably change the transcription levels of the auxin responsive genes *AUX1*, *AXR1*, *AXR2* and *SGT1B* confirming a role for TIR1 in induction of the PSR. Furthermore, application of Phi in Pi sufficient or deficient conditions to *tir1*-*1* did not affect the transcript levels of the auxin responsive genes tested with the exception of *AXR1* in 0.5 mM Phi-treated, Pi deficient plants (Figure 4). The results indicate that *ABA2* is to some extent required for both PSR induced auxin responsive genes and Phi induced auxin responsive gene expression. Likewise, *TIR1* is required for both PSR and Phi mediated auxin responsive gene expression, suggesting that Phi may act through mechanisms involving both ABA and auxin.

### Effects of Phi treatment on auxin-mediated root architecture

Given the potential of Phi to mimic the PSR in terms of auxin responsive gene expression, the potential for Phi to interfere with morphological responses of plant roots to Pi starvation was investigated.

**Figure 5:**
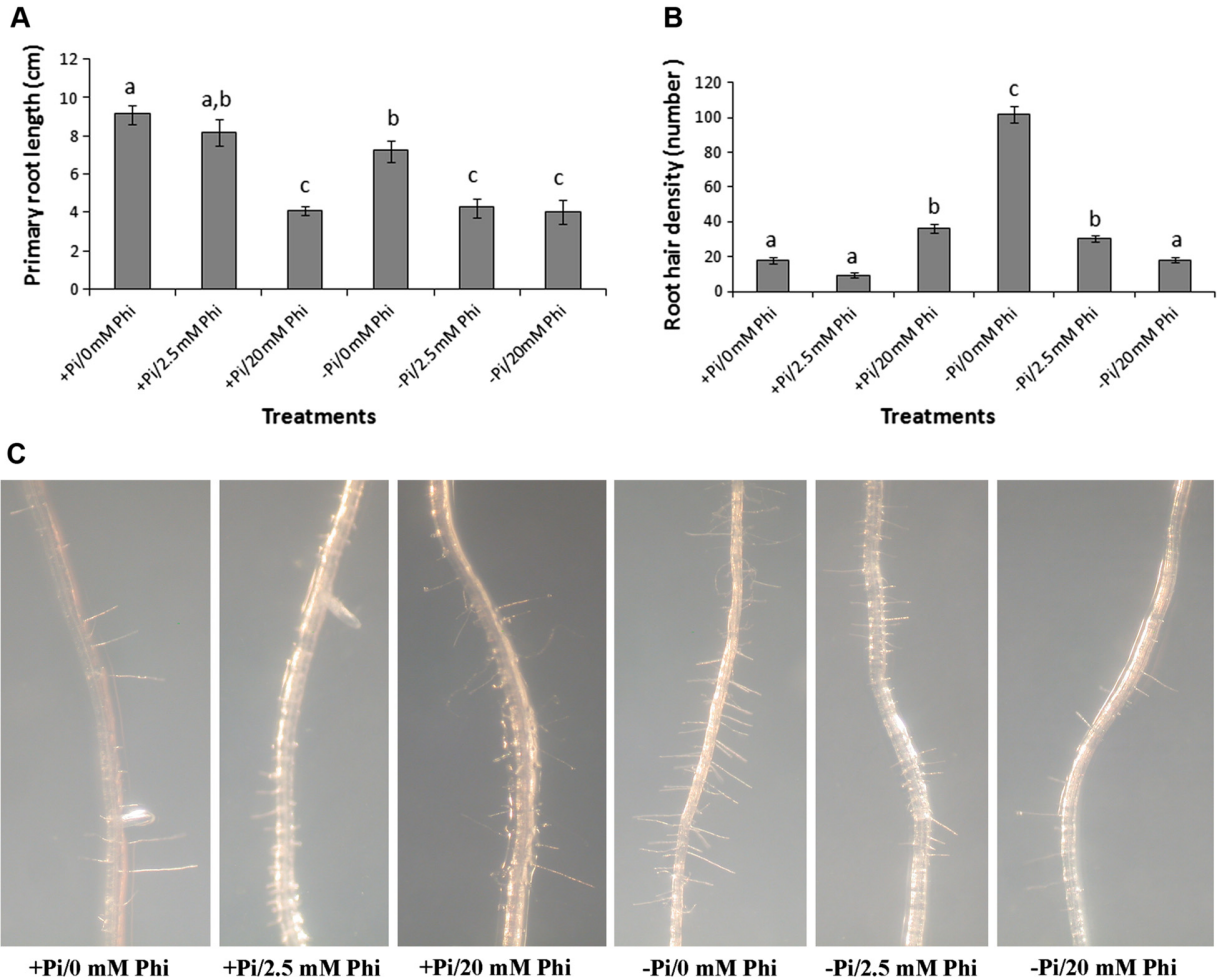
**Inhibitory effect of phosphite on primary root length and root hair density under phosphate deficiency.** The effect of phosphite (Phi) treatments on primary root length **(A)** and root hair density **(B)** of *Arabidopsis thaliana* ecotype Columbia (Col-0) grown under different phosphate (+Pi and -Pi) regimes. **(A)** Primary root length measured 7 days after transferring the seedlings to different Pi (+Pi; 1.25 mM and –Pi; 0 mM) and Phi media. Data are the means of 10 roots assessed per treatment with standard errors and bars with the same letter are not significantly different according to Tukey HSD test. **(B)** Root hair density (number) 7 days after transferring the seedlings to different Pi (+Pi and –Pi) and Phi medium. Root hair density was determined as the number of hairs in a 5 mm root segment, 2.5 mm from the root tip, and each bar represents the mean of six plants with standard error bars and bars with the same letter are not significantly (*P* > 0.05) different according to Tukey HSD test. **(C)** Shows the effect of different Phi concentration on root hair formation in seedlings grown in + Pi or –Pi media.

The primary root length of Pi starved Col-0 seedlings was significantly (*P* < 0.05) shorter than that in seedlings grown in Pi sufficient media (Figure 5A). Application of Phi resulted in suppression of primary root growth in seedlings grown in Pi sufficient media and the level of this suppression depended on the Phi concentrations used (Figure 5A). Pi starvation induced root hair formation in non-Phi treated seedlings compared to that observed in Pi sufficient grown Col-0 seedlings (Figure 5B, C). Furthermore, treatment of seedlings with a low concentration of Phi (2.5 mM) resulted in suppression of root hair formation in Pi sufficient grown seedlings, while in 20 mM Phi-treated seedlings root hair density was increased (Figure 5B,C). Phi at both 2.5 mM and 20 mM concentrations inhibited root hair formation induced by Pi starvation.

## Discussion

The findings of this study supported the role of auxin signalling in the induction of resistance to the predominantly necrotrophic pathogen *P. cinnamomi*. We further illustrate the effect of Phi on Pi signalling and the importance of their concomitant effect on activation/suppression of the auxin response pathway in relation to PSR.

### Phosphate starvation response mutant showed susceptibility to *P. cinnamomi*

The involvement of Phi in resistance to *P. cinnamomi*[53] and its interference in the phosphate starvation responses [15,54,55] suggested a possible role of Pi signalling in the outcome of *A. thaliana*―*P. cinnamomi* interactions. QPCR analysis of infection revealed that *phr1*-*1*, a mutant defective in response to Pi starvation was highly susceptible to *P. cinnamomi*; while, the mutants *pho2*-*1* and *pho1*-*2* remained resistant. The MYB-like transcription factor encoded by the *PHOSPHATE STARVATION RESPONSE 1* (*PHR1*) is vital for adaptation to phosphate deficiency in *Arabidopsis*[56] and this gene contributes to downstream Pi signalling by regulating the expression of Pi-responsive genes [38-40]. The PHO2 in *Arabidopsi*s is a sub-component of the Pi-signalling network that functions downstream of *PHR1* and regulates a subset of Pi-dependent responses, including Pi allocation between the shoot and the root [43,44]. Thus, mutation in the *PHR1* gene impairs many Pi signalling-related functions [38], while *pho1* and *pho2* mutations individually attenuate Pi uptake and distribution within tissues [57,58]. Up-regulation of *PHO1* is shown to be dependent on the *PHR1* transcription factor [41,42]. Furthermore, the cross talk between Pi, ABA and auxin signal transduction pathways have been suggested by Ribot et al. [42] demonstrating that application of exogenous ABA and auxin down-regulates the expression of *PHO1* independent of the plants’ Pi status [42]. Therefore, mutation of *PHR1* is likely to affect other Pi responses in addition to those dependent on PHO1 and PHO2 and the regulation of these responses, and resistance to *P. cinnamomi*, may be associated with ABA and/or auxin signalling.

### Auxin response pathway is involved in *P. cinnamomi* resistance

The phytohormone auxin, in addition to being involved in many aspects of development and growth in healthy plants [59-62], plays an important role in plant–pathogen interactions [9,63]. Together the role of the auxin (IAA) signalling pathway in the PSR [19], plant disease resistance [35] and the high susceptibility of *phr1*-*1* observed in the present study suggested a possible involvement of auxin signalling in resistance to *P. cinnamomi*. The QPCR analysis of *P. cinnamomi* infection in inoculated leaves of Col-0 and auxin-related mutants showed that *tir1*-*1*, an auxin receptor mutant was highly susceptible to *P. cinnamomi*. An effective auxin response in *Arabidopsis* depends on the removal of AUX/IAA family of transcriptional factor (TF) repressors through auxin-stimulated binding by the SCF^TIR1^ complex [46,48] and the TIR1 protein acts as an auxin receptor which directly links auxin perception to degradation of the AUX/IAA repressor proteins. In *Arabidopsis* auxin response mutants, the defective degradation of AUX/IAA transcriptional repressor proteins affect the induction of Auxin Response Factors (ARFs) and consequently the expression of auxin responsive genes [64]. Therefore, the susceptibility of the *tir1*-*1* mutant which is defective in the F-box TIR1 protein and AUX/IAA degradation [45-47] suggested that ubiquitin-mediated AUX/IAA protein degradation is important in plant resistance to *P. cinnamomi*.

TIBA (a polar auxin transport inhibitor) treatment also led to the enhanced susceptibility of lupin seedlings to *P. cinnamomi* suggesting that the suppression of auxin transporters and consequently distruption of auxin signalling is important in plant resistance to *P. cinnamomi*. Llorente et al. [35] also suggested the involvement of the auxin signalling pathway in resistance to necrotrophic pathogens by demonstrating that the suppression of the auxin response pathway enhanced the susceptibility of *Arabidopsis* to *Botrytis cinerea* and *Plectosphaerella cucumerina*.

In *Arabidopsis*, SGT1B contributes to the auxin response controlled by the SCF^TIR1^ complex [30,45], through SCF-TIR1 mediated degradation of AUX/IAA repressor proteins [29-31]. SGT1B also functions in *R* gene mediated plant disease resistance signalling and in this regard interacts with RAR1 [29,33]. When challenged with *P. cinnamomi* the *sgt1b*-*1* mutant showed no significant (*P* > 0.05) difference to its parental background Col-0, suggesting that SGT1B contributes a redundant role to resistance to *P. cinnamomi*. Together the susceptibility of *tir1*-*1*, the enhanced susceptibility of TIBA-treated plants and the resistance of *sgt1b*-*1* indicated that auxin plays a substantial role in resistance to *P. cinnamomi* through a SCF^TIR1^-mediated ubiquitination mechanisim that is independent to SGT1B function.

### Involvement of 26S proteasome in *A. thaliana*–*P. cinnamomi* interaction

The 26S proteasome is involved in the ubiquitination of AUX/IAA proteins and consequently activation of auxin responsive genes [49], and mutants that are compromised in 26S proteasome activity attenuate auxin sensitivity and other plant processes such as root apical meristems maintenance, leaf organ size and gametophyte developments [28,65-68]. In the present study, several *Arabidopsis* mutants defective in 26S proteasome subunits (*pbe1*, *rpt2a*, r*pt2b*, *rpt5a* and *rpn10*) were screened for their susceptibility to *P. cinnamomi*. The analysis of pathogen infection revealed that *pbe1*, a 20S proteasome knockout mutant was highly susceptible to *P. cinnamomi* in comparison to its parental background Col-0. In addition, the *rpn10* mutant, defective in ubiquitin/26S proteasome-mediated proteolysis in auxin and ABA signalling, was susceptible to *P. cinnamomi*. RPN10 is a subunit of the 26S proteasome pathway which affects several regulatory processes in *Arabidopsis* by directing the unwanted proteins to the 26S proteasome for degradation [51]. The *Arabidopsis rpn10* mutant shows a decreased sensitivity to auxin and is highly sensitive to exogenous application of ABA [51]. The reduction in auxin sensitivity in *rpn10* may relate to its susceptibility to *P. cinnamomi* and further supports a role for TIR1/26S proteosome in resistance to *P. cinnamomi*.

Furthermore, *Arabidopsis* 26S proteasome subunit mutants *rpt5a*, *rpt2a* and *rpt2b* (homologue of *rpt2a*) also showed a higher level of susceptibility compared to that observed in their parental background Col-0. The susceptibility of the mutants defective in 26S proteasome subunits to *P. cinnamomi* suggested a role of 26S proteasome subunits in resistance to *P. cinnamomi*, possibly through degradation of auxin inhibitor proteins following their ubiquitination by TIR1.

### Concomitant effect of Phi and Pi is relevant for the activation/suppression of PSR- and auxin-related genes

The role of Phi in induction of resistance to *Phytophthora* has been demonstrated in several studies suggesting its complex mode of action including (i) acting directly by inhibition of pathogen growth, (ii) acting indirectly by inducing the release of stress metabolites from the pathogen to elicit the defence response and (iii) indirectly stimulating host defence responses [53,69-72]. Eshraghi et al. [53] found that Phi mediated resistance to *P. cinnamomi* in the susceptible *Arabidopsis* ecotype Ler resembled the response of the resistant ecotype Col-0 in terms of timing and the defence responses induced. Similar observations for Phi mediated resistance were reported for *P. infestans*-challenged potato [73] and *P. palmivora*-challenged *A. thaliana*[71]. Previous research demonstrated that Phi primed some aspects of the defence response, such as the expression of defence genes involved in the SA, JA/ET pathways in the absence of a pathogen [53,71,73]. However, screening SA and JA/ET related knockout mutants in the presence/absence of Phi suggested that Phi mediated resistance to *P. cinnamomi* in *A. thaliana* was independent of the SA, JA or ET signalling pathways [52].

Phi has also been shown to interfere with a broad range of biochemical and developmental responses including PSR in plants [15-18] many of which have been shown to rely on auxin signalling involving the SCF^TIR1^ UPP complex [19-21]. The susceptibility of *Arabidopsis* auxin response pathway mutants and the Pi response mutant *phr*-*1* to *P. cinnamomi* in this study together with the interference of Phi in Pi homeostasis and its role in the induction of plant defence responses against *P. cinnamomi*[53] suggested that Phi mediated resistance could be through its’ effect on Pi signalling, and in particular, on the auxin signalling pathway.

The transcript levels of the PSR responsive genes *AtPT2*, *AtACP5*, and *AT4* increased in response to Pi deficiency. However, Phi applications at all levels suppressed their enhanced expression similar to that observed in plants grown in Pi sufficient media, demonstrating the effect of Phi in suppression of PSR. These results are supported by Ticconi et al. [54] who reported a similar effect of Phi on suppression of PSR genes. While *PHR1* expression was increased by Phi treatment of Col-0 under Pi sufficient conditions, similar increases in expression of *AtPT2*, *AtACP5* and *AT4* were not observed. This may be because *PHR1* contributes to downstream Pi signalling by regulating the expression of Pi responsive genes [38,40] and *PHO1* and *PHO2* both act downstream of the *PHR1* transcription factor to control the local uptake or Pi allocation between the shoot and the root involving *AtPT2*, *AT4* and *AtACP5*[41,44,74]. Although the mutation in the *PHR1* gene impairs many Pi signalling-related functions, the studies by Ribot et al. [42] suggested that the expression of *PHO1* is independent of the plants’ Pi status. Therefore, PHR1 is likely to affect other Pi responses in addition to those dependent on PHO1 and PHO2 and the regulation of these responses.

Perez-Torres et al. [25] demonstrated that auxin sensitivity was enhanced in Pi deficient *Arabidopsis* plants largely through increased expression of *TIR1*, which accelerated the degradation of AUX/IAA proteins. In addition to the potential interaction with PSR/auxin signalling described above, Eshraghi et al. [52] suggested that Phi acts partially through an ABA dependent mechanism. Therefore, to investigate whether Phi acts through *TIR1* or *ABA2* and whether mutations in these two genes are affecting the impact of Phi on PSR, the effect of Phi on expression of *AT4* and *PHR1* at the transcriptional level was further tested in Pi sufficient and Pi deficient grown Col-0, *aba2*-*4* and *tir1*-*1*. Although in Col-0 Phi treatments suppressed the enhanced transcript levels of *PHR1* induced by Pi deficiency, Phi enhanced the transcript levels of *PHR1* in Pi sufficient grown samples and the level of this induction depended on the Phi concentrations used. These results suggested that although Phi suppressed the PSR in Pi starved plants, application of Phi to Pi sufficient plants resulted in activation of PSR. One explanation for this may be competition between Pi and Phi for uptake or transport. It has been shown in *Brassica* spp. that high Phi concentrations inhibit plant development by competing with Pi absorption [75,76]. Our results showed that the expression of *PHR1* in response to Pi starvation was affected in the *aba2*-*4* and *tir1*-*1* mutants suggesting the importance of *ABA2* and *TIR1* genes in the induction of *PHR1* and PSR. Furthermore, Phi treatments had no considerable effect on transcript level of the *PHR1* gene in either *aba2*-*4* or *tir1*-*1* mutants suggesting that mutation in the *ABA2* and *TIR1* genes may disrupt the Phi effect on Pi signalling.

The induction of the *PHR1* gene by Phi in Col-0 samples grown in Pi sufficient media suggested that Phi induces PSR and the loss of *PHR1* gene expression in the *tir1*-*1* mutant highlighted the possible induction of the auxin response pathway by Phi treatments. To test this further, the concomitant effect of Phi and Pi on auxin signalling in Pi sufficient/Pi deficient grown Col-0, *aba2*-*4* and *tir1*-*1* mutants was assessed by measuring the relative expression ratios of the auxin responsive genes, *AUX1*, *AXR1*, *AXR2* and *SGT1B* transcripts. Pi deficiency increased the transcript levels of all genes tested suggesting the induction of the auxin response pathway in Pi starved plants. In Pi sufficient Col-0, Phi treatments (2.5 and 20 mM) induced the transcript levels of *AUX1*, *AXR1*, *AXR2* and *SGT1B* and the level of this induction depended on the Phi concentrations used. Moreover, Pi starvation did not considerably change the transcription levels of the auxin responsive gene *AUX1*, *AXR1*, *AXR2* and *SGT1B* in the *aba2*-*4* and *tir1*-*1* mutants confirming a role for *ABA2* and *TIR1* in the induction of the PSR. Overall, the results suggested that *ABA2* and *TIR1* genes are required for both PSR and Phi mediated auxin responsive gene expression, indicating that Phi may act through both the ABA and auxin pathways.

### Effect of Phi on root morphology

Considering the potential of Phi to mimic the PSR in terms of auxin responsive gene expression, the effect on morphological responses to Pi starvation was investigated, as Pi status acting through auxin signalling is important for determining root architecture [19-21]. Pi deficiency suppressed the primary root length and induced root hair formation in roots of ecotype Col-0 and the application of Phi resulted in suppression of primary root growth in seedlings grown in either Pi sufficient or Pi deficient media in a dose–response manner. The morphological responses of Pi starved roots were consistent with those previously described [20,21,77-79]. Phi at lower concentration (≤ 2.5 mM) inhibited root hair formation induced under phosphate starvation; however, 20 mM Phi induced root hair formation in Pi sufficient plants. Gilbert et al. [80] also showed that Phi dramatically increased the number of proteoid root segments (a phosphate starvation response) in Pi sufficient lupin seedlings. Overall, both, morphological and gene expression data suggested the involvement of the auxin signaling pathway and phosphate signalling in responses to Phi treatment.

## Conclusions

This study highlighted the importance of Pi signalling in plant resistance to *P. cinnamomi* by illustrating the susceptibility of *phr1*-*1* (a mutant defective in Pi signalling) and linked this role with the auxin response pathway through the susceptibility of *tir1*-*1* and TIBA-treated plants to *P. cinnamomi*. A role for the 26S proteasome, which is required for auxin signalling [28,49], was further supported by the susceptibility of lines with mutations in various components. Moreover, the link between Phi treatment and PSR, as demonstrated by morphological PSR responses and analysis of Pi starvation gene expression following Phi treatment under Pi sufficient and deficient conditions and in auxin and ABA response mutants, suggested that the mechanism of action of Phi may include modulation of Pi signalling involving auxin.

## Methods

### Plant and pathogen materials

*Arabidopsis thaliana* accession Columbia (Col-0) and several *A. thaliana* mutant/transgenic lines (Table 2) in the Col-0 background that are defective in different signalling pathways were used in this study. *Arabidopsis thaliana* genotype Col-0 was purchased from LEHLE Seeds (Round Rock, TX), and the mutants *rpt2a*, *rpt5a*, *rpn10*, and *cni1* were provided by Dr Derek Gotto and Prof. Junji Yamaguchi (Hokkaido University, Japan). The remaining mutants were obtained from the *Arabidopsis* Biological Resource Centre (ABRC, Ohio State University); https://abrc.osu.edu/. Blue lupin (*L. angustifolius* L., cv. Mandalup) seeds were obtained from Department of Agriculture and Food, Western Australia (DAFWA).

**Table 2:** **The list of*Arabidopsis*mutant**/**transgenic lines used in this study**

**Name**	**Locus**	**Genetic alteration**	**Phenotypes**	**References**
*aba2*-*4*	AT1G52340	EMS mutant	Defective in ABA biosynthesis, reduced sensitivity to sugar and glucose	[86]
*phr1*-*1*	AT4G28610	T-DNA-insertion	Defective in response to phosphate (Pi) starvation	[74,87]
*pho1*-*2*	AT3G23430	EMS mutant	Decreased Pi level in shoot, but normal Pi level in root	[88]
*pho2*-*1*	AT2G33770	EMS mutant	Pi over-accumulator and exhibits increased levels of Pi in the shoots	[89]
*tir1*-*1*	AT3G62980	EMS mutant	Defective in auxin response	[90]
*sgt1b*-*1*	AT4G11260	EMS mutant	Defective in SCF^TIR1^ mediated auxin response	[30]
*pbe1*	AT1G13060	T-DNA-insertion	A knockout mutant for 20S proteasome	[50]
*rpt2a*	AT4G29040	T-DNA-insertion	Defective in 26S proteasome subunit	[65,68,91]
*rpt2b*	AT2G20140	T-DNA-insertion	Defective in 26S Proteasome Subunit	[65,91]
*rpt5a*	AT3G05530	T-DNA-insertion	Defective in 26S Proteasome Subunits	[68]
*rpn10*	AT4G38630	T-DNA-insertion	Defective in ubiquitin/26S proteasome-mediated proteolysis (UPP) substrate recognition and in abscisic acid signalling	[51,92]

All lines mentioned are in the Col-0 background.

*Phytophthora cinnamomi* (isolate MP 94.48) was obtained from the Centre for *Phytophthora* Science and Management (CPSM) at Murdoch University. *Phytophthora cinnamomi* zoospores were produced aseptically according to the method described by Byrt & Grant [81], and the zoospores density was determined using a bright line haemocytometer and adjusted to a concentration of 1 × 10^5^ zoospores mL^−1^ using sterile distilled water.

### Plant growth conditions and inoculation procedure

*Arabidopsis thaliana* ecotype Col-0 and mutants were germinated on half strength Gamborg's B-5 Basal medium with 0.8% (w/v) phytagar [82]. In addition, 2.5 mM MES [2-(N-morpholino)-ethanesulphonic acid]-KOH (pH 5.7) and 0.5% (w/v) sucrose were included (pH 5.7, adjusted with KOH). After sowing the seed on the medium, seeds were stratified for 3 days at 4°C in the dark before being transferred to a growth cabinet at 21°C ± 1°C with a 10-h photoperiod at a photon fluorescence rate of 100 μmol m^−2^ s^−1^. To study *A. thaliana*―*P. cinnamomi* interactions, attached leaves of four-week-old seedlings were inoculated either with 3 μL of 1 × 10^5^*P. cinnamomi* zoospores mL^−1^ or 3 μL of sterile distilled water (control) on the abaxial surface. Five samples per genotype were collected 72 h after inoculation for quantitative PCR (QPCR) analysis of infection and the experiment was conducted twice.

The lupin (*Lupinus angustifolius* L., cv. Mandalup) seeds were surface-sterilized in 70% ethanol for 2 min followed by immersion in 50% bleach solution (6.25% available chlorine) for 5 min. The sterilized seeds were germinated on sterile filter paper pre-moistened with distilled water at 25°C in the dark for 3 days. The seedlings were placed on a bed of damp absorbent paper (24 × 38.5 cm), placed between two layers of clear plastic, rolled up and placed in 200 mL beakers filled with 50 mL half-strength hydroponic Hoagland medium [83] and grown for a further five days in a growth cabinet with a 10-h photoperiod (100 μmol m^−2^ s^−1^ at 21 ± 1°C) until treatments. Lupin seedlings were inoculated by placing a 4 mm diameter plug of *P. cinnamomi* mycelium at the tips of roots. Root tissue samples were collected for lesion size assessments at 48 h and 72 h after inoculation.

### Quantitative PCR analysis of infection

To determine the level of infection quantitatively, QPCR analysis was conducted and the relative amount of *P. cinnamomi* biomass (DNA) in infected *Arabidopsis* leaf samples was measured and normalized based on plasmid DNA (internal control) according to Eshraghi et al. [37]. Samples were collected and snap frozen 72 h after inoculation. Five samples per treatment each containing four leaf discs (7 mm in diameter) from one seedling were collected.

### TIBA treatments and lesion size assessments in lupin

The auxin transport inhibitor TIBA (2, 3, 5-triiodobenzoic acid; Sigma Aldrich) was dissolved in absolute ethanol and filter sterilised TIBA was added to sterilized half-strength liquid Hoagland medium to give a final concentration of 10 μM. TIBA treatment was conducted on five-day-old lupin seedlings by transferring the seedlings to half-strength hydroponic Hoagland medium containing 10 μM TIBA (+TIBA). For controls (−TIBA), the seedlings were transferred to the half-strength hydroponic Hoagland medium with the same amount of ethanol as in + TIBA medium. 48 h after treatments, the roots were inoculated by placing a 4 mm diameter plug of *P. cinnamomi* mycelium at the root tips and seedlings were kept in half-strength hydroponic Hoagland medium until harvested.

A minimum of 10 lupin roots per treatment were assessed for lesion development and the experiment was repeated twice. The level of infection was measured 48 and 72 h after *P. cinnamomi* inoculation and the data were presented as mean percentage infected root area. The digital images of the roots were captured with as Epson Expression 1680 scanner and the area of lesions formed by *P. cinnamomi* infection were calculated using the program WinRHIZO™ (Régents Instruments, Inc.).

### Cloning PHR1 and TIR1

In order to restore function of *PHR1* and *TIR1* genes in *phr1*-*1* and *tir1*-*1* knock out mutants, these gene were cloned and transferred to the knock out mutants as follow. Col-0 genomic DNA was used as template for cloning *PHR1* gene (AT4G28610) and *TIR1*gene (AT3G62980). PCR was performed with Phusion® High-Fidelity DNA Polymerase (BioLabs) according to manufacturer’s instructions using primers containing the attB recombination sites (*PHR1*–forward 5´– GGG GAC AAG TTT GTA CAA AAA AGC AGG CT T *CTC TTC CTT GGT CCT GGA TTG* and *PHR1*–reverse 5´–GGG GAC CAC TTT GTA CAA GAA AGC TGG GTC *TCT TCC TTG GGG ATC TGT TG*, *TIR1*–forward 5´–GGG GAC AAG TTT GTA CAA AAA AGC AGG CTT *CCG CTG TCC AAC TTC TTC CTC* and *TIR1*–reverse 5´–GG G GAC CAC TTT GTA CAA GAA AGC TGG GTC *GTT CCT AAA CCG GAA CAC GA*. The PCR products were cloned to pDONR207 (Invitrogen) using Gateway® BP Clonase® II Enzyme Mix (Invitrogen) according to the manufacturer’s instructions and transformed into *E. coli* competent cells (DH5α). After confirmation by sequencing, the insert was cloned into the Gateway® compatible expression vector pGREEN0179 containing CAMV 2x35S promoter and CAMV terminator with a Gateway A cassette [84] using Gateway® LR Clonase® II enzyme mix (Invitrogen) according to manufacturer’s instructions. Positive clones were confirmed by PCR and *phr1*-*1*, and *tir1*-*1* mutants were transformed using *Agrobacterium tumefaciens* C58C1 (pCH32) [85].

### Quantitative measurements of gene transcription using qRT-PCR

For quantitative measurement of gene transcription, seeds of ecotype Col-0 and the *A. thaliana* mutants *aba2*-*4* and *tir1*-*1* were germinated on half strength 0.8% (W/V) phytagar-Gamborg B-5 basal medium (pH 5.7) as previously described. The seedlings were grown in a growth cabinet at 21 ± 1°C with a 10-h photoperiod at a photon fluorescence rate of 100 μmol m^−2^ s^−1^ for three weeks and then transferred to half strength 0.8% (W/V) phytagar—Hoagland medium [83] (pH 5.7, adjusted with KOH) with different concentrations of Pi (0 and 1.25 mM) and Phi (0, 0.5, 2.5 and 20 mM) in a completely randomized design and grown for a further five days. A stock solution of filter sterilized potassium Phi (pH 5.7, adjusted with KOH) was freshly prepared from phosphorous acid (Aldrich Chemicals) and mixed with sterilized medium to give the specified Phi concentration. Samples were collected, frozen in liquid nitrogen and stored at −80°C until RNA extraction and qRT-PCR.

Four biological samples per treatment were randomly collected for gene expression analysis and each sample was a pool of three plants. Tissue samples were homogenized using Tissuelyser® (Qiagen, Inc.) and the RNeasy plant mini kit (Qiagen, Inc.) was used to isolate RNA according to the manufacturer’s instructions. Approximately, 3 μg of DNA-free RNA was used for first-strand cDNA synthesis using the SuperScript III First-Strand Synthesis System for RT-PCR (Invitrogen). Gene specific primers (Additional file 1: Table S1) were designed using Primer Express 1.5 software (Applied Biosystems Inc.) and qRT-PCR conducted according to [53]. The transcript levels of all genes in the mutants were normalized based on expression of actin 2 (*ACT2*) measured in the same samples and presented relative to the normalized expression levels in corresponding Pi sufficient, non-Phi-treated plants as fold expression.

### Root morphology assessments

For morphological assessments of *A. thaliana* roots, seeds of ecotype Col-0 were grown on half strength Gamborg B-5 basal medium with 0.8% (W/V) phytagar (pH 5.7) as previously described. One-week-old seedlings were transferred to half strength Hoagland medium with 0.8% (W/V) phytagar [83] (pH 5.7) with different concentrations of Pi (0 and 1.25 mM) and Phi (0, 2.5 and 20 mM) in a completely randomized design and grown for further seven days. A minimum of 10 seedlings per treatment were assessed for their primary root growth using the photographs taken by a digital camera (Nikon; Cool PIX 995) and the experiment was repeated twice. A minimum of six seedlings per treatment were assessed for their root hair density using the photographs taken by a binocular microscope (Olympus SZ40) with an attached digital camera (Nikon; Cool PIX 995) and the experiment was repeated twice. Root hair density was determined as the number of hairs in a 5 mm root segment (from the root tip).

### Data analysis

ANOVA was used in all measurements to compare the treatments and the normality of residuals was tested using GenStat software (14th edition). 5% least significant difference (LSD) was calculated for the mean comparisons of treatments and genotypes using GenStat software (14th edition).

## Abbreviations

PSR: Phosphate starvation response; ABA: Abscisic acid; SA: Salicylic acid; JA: Jasmonic acid; ET: Ethylene; IAA: Indole-3-acetic acid; TIBA: 2, 3, 5-triiodobenzoic acid; UPP: Ubiquitin proteasome pathway; qRT-PCR: Quantitative reverse transcription polymerase chain reaction; QPCR: Quantitative real-time polymerase chain reaction.

## Competing interests

The authors declare that they have no competing interests.

## Authors’ contributions

LE carried out the experimental designs, disease assessments, gene expression studies, cloning, transformations, data analysis, and drafted the manuscript. JA and NA participated in the design of the gene expression experiments, disease screening assessments, and involved in the interpretation of data and revising the manuscript. JM and BS participated in the design of disease assessment assays and revising the manuscript. GH coordinated the study and involved in interpretation of disease assessments and critically revising the manuscript. All authors read and approved the final manuscript.

## Supplementary Material

Additional file 1: Table S1 Sequences of the gene-specific primer pairs used in quantitative real-time reverse transcription polymerase chain reaction (qRT-PCR) experiments. Click here for file
