# Supplementary material for: Suppression of the auxin response pathway enhances susceptibility to Phytophthora cinnamomi while phosphite-mediated resistance stimulates the auxin signalling pathway
Source: BMC Plant Biol. 2014 Mar 20;14:68. doi: 10.1186/1471-2229-14-68 (PMC3999932; doi:10.1186/1471-2229-14-68)
Supplement: Additional file 1: Table S1 — Sequences of the gene-specific primer pairs used in quantitative real-time reverse transcription polymerase chain reaction (qRT-PCR) experiments. [file 1471-2229-14-68-S1.docx]

**Additional file 1: Table S1** Sequences of the gene-specific primer pairs used in quantitative real-time reverse-transcription polymerase chain reaction (qRT-PCR) experiments

| Gene^a^ | AGI number | Forward and reverse primers (5´– 3´) |
| --- | --- | --- |
| *AT4* | [AT5G03545](http://www.arabidopsis.org/servlets/TairObject?id=1000429503&type=locus) | GTGTGTGAATGGAGCGATGAA  GATCGAAGTTGCCCAAACGA |
| *AtACP5* | AT3G17790 | GGTGACGCAGAAGCTCAGCT  CCAACTCTGCATCAACGACAA |
| *AtPT2* | [AT2G38940](http://www.arabidopsis.org/servlets/TairObject?id=35340&type=locus) | GTTTTGGCTTGGATTTGGCA  AAAGGCTCCGCGAGTCTTCT |
| *PHR1* | [AT4G28610](http://www.arabidopsis.org/servlets/TairObject?id=130090&type=locus) | CAAATTCCGCAACCTCAGAT  CCGTTCCGTTATTGCTGTTT |
| *AUX1* | AT2G38120 | CGCTGTTATCCTCATGCTCA  GCATCCCAATCACTTTCTCC |
| *AXR1* | AT1G05180 | ATCGTCAGCTCAGGATTTGG  CAAAGCCTCGGAACCAGTAG |
| *AXR2* | AT3G23050 | TCTCCGAAACCGTTGATCTC  CTTCTCCTTGGGAACAGCAG |
| *SGT1B* | AT4G11260 | TGCTCCTCCTGTTCCAATTC  ATTGTCACCACCGCTTCTTC |
| *ACT2* | AT3G18780 | CTTGCACCAAGCAGCATGAA  CCGATCCAGACACTGTACTTCCTT |

*AT4*: *AtIPS2* (induced by Pi starvation 2); *AtACP5*: *Arabidopsis thaliana* acid phosphatase 5; *AtPT2: Arabidopsis thaliana* phosphate transporter 2; *PHR1*: phosphate starvation response 1; *ABA3*: abscisic acid (ABA) deficient 3; *AUX1*: auxin resistant 1; *AXR1*: auxin resistant 1; *AXR2*: auxin resistant 2; *SGT1B*: enhancer of *tir1-1* auxin resistance 3; and *ACT2*: actin 2.
